# Editorial Comment: Association between selfreported mobile phone use and the semen quality of young men

**DOI:** 10.1590/S1677-5538.IBJU.2024.9904

**Published:** 2024-03-18

**Authors:** Pedro Henrique Peixoto Costa, Arthur Cardoso Del Papa, Arie Carneiro

**Affiliations:** 1 Hospital Israelita Albert Einstein Departamento de Urologia São Paulo SP Brasil Departamento de Urologia, Hospital Israelita Albert Einstein, São Paulo, SP, Brasil;; 2 Centro Universitário -FMABC Departamento de Urologia Santo André SP Brasil Departamento de Urologia, Centro Universitário -FMABC, Santo André, SP, Brasil

Rita Rahban 1, Alfred Senn 2, Serge Nef 2, Martin Röösli 3

**Fertil Steril. 2023 Dec;120(6):1181-1192.**

DOI: 10.1016/j.fertnstert.2023.09.009 | ACCESS: 37921737

## COMMENT

The paper by Rahban et al. is a significant observational study that analyzes the influence of mobile phone on semen parameters. It is a population-based study that included a sample of 2886 Swiss men aged between 18 and 22 years, undergoing military enlistment from 2005 to 2018 [1].

As mentioned by the authors, semen quality has been significantly deteriorating in recent decades without a clear definition of possible causes. With the increase in mobile phone use, leading to greater exposure to radiofrequency electromagnetic fields (RF-EMFs), as well as changing lifestyle patterns, studies investigating environmental factors and habits that may be related to seminal quality and fertility are crucial.

The authors associated a decreasing sperm concentration and total sperm count (TSC) with increased frequency of mobile phone use. There was no negative correlation with other semen parameters or with the position of the phone when not in use.

In our opinion, the challenge lies in separately studying potential confounders for these findings. Previous studies have reported the association between worsening semen parameters and factors such as diet [2], caffeine consumption [3], sedentarism [4], occupational exposure [5], use of medications and other drugs, cannabis [6], and exogenous testosterone [7]. The authors conducted a linear regression model to adjust for confounding factors (BMI, alcohol consumption, smoking, and others), and the findings were consistent. However, the study itself observed that men with higher mobile phone use had a higher proportion of smoking and alcohol consumption, higher BMI and medication consumption, reported less good or excellent health, and lower educational levels.

Therefore, the association of these factors contributing to a less healthy lifestyle seems evident, and mobile phone use may be negatively impacting people’s lives. Studies, especially those conducted in children and adolescents, have shown that individuals with more screen time and mobile phone use are often more sedentary [8, 9], have a tendency towards obesity and sleep disorders [10], and are subject to greater mental health problems, mainly related to social media use [11].

Also, understanding the impact of RF-EMFs on spermatogenesis necessitates precise data on the duration, intensity, and particularity of exposure. This study relies on self-reported usage frequency rather than accurate measurements of exposure duration. This approach fails to capture critical nuances such as varying usage patterns (continuous versus intermittent), time of use, differences in cell phone models, distance from the body, use of hands-free devices, signal strength, and other factors that can significantly affect RF-EMF exposure. Such biases limit the reliability of conclusions drawn about the relationship between RF-EMF exposure and sperm health.

While cell phones are a primary concern due to their ubiquitous use, it is essential to recognize that other electronic devices also emit RF-EMFs. Wi-Fi routers, computers, TVs, radio, tablets and various wireless gadgets contribute to overall exposure, complicating efforts to isolate the specific impact of cell phone RF-EMFs on spermatogenesis. This multifaceted exposure landscape requires a comprehensive approach to understand its collective influence on male reproductive health.

In conclusion, the study addresses a very relevant topic and has a substantial number of participants. However, the methodology and study design do not allow us to reach a conclusion to provide specific guidance. The study raises an important hypothesis and alerts the scientific community that excessive mobile phone use may correlate with a decline in fertility, emphasizing the importance of prospective observational studies to assess the consequences of RF-EMF exposure and the impact of mobile phone and other technology use on men’s quality of life and fertility.
